# Caregiver burden among caregivers of patients with depression: a systematic review and meta-analysis

**DOI:** 10.3389/fpsyt.2026.1878799

**Published:** 2026-08-05

**Authors:** Xu Huang, Ruo Zhou, Huijuan Lei, Jiale Li, Qian Ye, Yiling Yang, Tianhui You

**Affiliations:** 1 School of Nursing, Guangdong Pharmaceutical University, Guangzhou, Guangdong, China; 2 School of Health and Nursing, Guangzhou Huali College, Jiangmen, Guangdong, China; 3 School of Continuing Education, Guangdong Pharmaceutical University, Guangzhou, Guangdong, China

**Keywords:** depression, informal caregiver burden, informal caregivers, major depressive disorder, meta-analysis, systematic review

## Abstract

**Background:**

Depression is a highly prevalent mental disorder with a global distribution. Informal caregivers are integral to the long-term care of individuals with depression. However, they frequently experience substantial caregiver burden, detrimentally impacting both caregiver well-being and patient prognosis. Despite the clinical significance of this issue, there is currently no systematic review or meta-analysis quantifying the overall magnitude of this burden and synthesizing its associated factors among informal caregivers of patients with depression.

**Objective:**

This study aimed to assess the level of caregivers’ burden among informal caregivers who are taking care of patients with depression and to systematically evaluate the associated factors through a narrative synthesis guided by the Stress-Appraisal Model.

**Methods:**

Eight electronic databases (PubMed, MEDLINE, Embase, PsycINFO, Web of Science, CNKI, Wan fang, and VIP) were systematically searched. Data extraction and quantitative synthesis were performed using STATA version 18.0. Statistical heterogeneity was assessed via Cochran’s Q test and the *I*^2^ statistic, followed by robust sensitivity and subgroup analyses. Concurrently, a narrative synthesis guided by the Stress-Appraisal Model was conducted to systematically identify associated factors, providing a theoretical basis for future targeted interventions.

**Results:**

The current systematic review and meta-analysis comprised all 1366 research respondents from 11 studies. The overall pooled mean score of caregiver burden was 38.15 (95% CI: 31.38, 44.92; *I*^2^ = 98.9%, *p* < 0.001) with a 95% prediction interval ranging from 19.67 to 56.67. Subgroup analyses indicated no significant differences based on patient type or country. However, caregivers of patients with severe depression exhibited a higher pooled mean burden 42.40 (95% CI: 29.85, 54.94) compared to those caring for patients with moderate depression 35.51 (95% CI: 22.74, 48.28). Furthermore, a disease course exceeding one year yielded a higher caregiver burden 39.26 (95% CI: 33.34, 45.18) than a duration of less than one year 34.42 (95% CI: 27.84, 40.99).

**Conclusion:**

The current systematic review and meta-analysis showed that caregivers of patients with depression generally experience substantial caregiving burden, with pooled effect estimates indicating that the overall severity of this burden approaches the moderate level. Nevertheless, due to the limited quantity of caregiving burden is affected by multiple clinical variables. Accordingly, the findings of current systematic reviews should be interpreted with caution. Furthermore, this study proposes that public health interventions targeting mental health indicators and social support systems should be implemented for informal caregivers of individuals with depression, with the objectives of reducing the caregiving burden of this population and potentially improving the quality of care accessed by patients with depression.

**Systematic review registration:**

https://www.crd.york.ac.uk/PROSPERO/display_record.php?ID=CRD420261373459, identifier CRD420261373459.

## Introduction

Depression affects approximately 322 million individuals globally, representing 4.4% of the world population and constituting a leading cause of disability worldwide (1). Although fundamentally classified as an individual mood disorder, the protracted and recurrent trajectory of depression, coupled with associated functional impairments, frequently establishes it as a complex dyadic challenge involving both patients and their families (2, 3).

As healthcare systems transition toward community and home-based psychiatric management, the primary responsibility for daily care has increasingly shifted from formal medical institutions to informal family caregivers (4). Consequently, informal caregivers are subjected to chronic, multidimensional demands that are frequently associated with adverse physical, psychological, and socioeconomic outcomes (5, 6). Despite extensive documentation of caregiving distress, health-risk behaviors, and clinical burnout in general medical contexts, the specific burden associated with depression caregiving has received substantially less academic attention compared to neurodegenerative or oncological diseases (7). Specifically, caregiver burden in the context of depression remains underexplored, particularly within settings characterized by high familial dependence and limited healthcare resources (8).

The concept of caregiver burden covers the multifaceted stress experienced during the process of caring for sick relatives or close contacts, and informal caregivers mainly refer to parents, spouses and children (9). This burden is conventionally categorized into objective domains, such as the disruption of family relationships, constraints on social and occupational activities, and negative associations with physical health, and subjective domains, which involve psychological reactions including sadness, anxiety, relational frustration, and the emotional strain of coping with disturbing clinical manifestations (10). Empirical literature indicates that the most frequent correlates of caregiver burden are patient symptom severity and illness-related functional deficits (11). Additional associated factors include disease duration, daily caregiving hours, co-residence status, illness-related social stigma, family dynamics, and a history of patient self-harm or aggressive behaviors (12–14).Since its development, the standardized Zarit Burden Interview (ZBI) has been extensively adopted in chronic disease research in chronic disease research and has gradually been extended to psychiatric populations (15). However, it is imperative to acknowledge that the clinical manifestations of depression might aggravate caregiving challenges through distinct interpersonal mechanisms that warrant tailored investigation.

The clinical complexity of depression makes informal caregiving uniquely demanding. Unlike purely physical or motor disabilities, depression involves an intricate interplay of affective, cognitive, and behavioral symptoms that can significantly complicate the caregiving experience (16–22). For instance, disease-specific manifestations such as severe apathy, emotional withdrawal, and irritability directly impair interpersonal communication within the caregiving dyad (23). Caregivers might misinterpret a patient’s clinical withdrawal as personal rejection or uncooperativeness. It is plausible that this absence of emotional reciprocity contributes to relational isolation, psychological distress, and caregiving frustration. Informal family caregivers, predominantly comprising spouses, adult children, and parents, constitute the foundational pillar of mental health support systems (24). Empirical evidence suggests that caregiver burden associated with depression is substantially elevated compared to normative population levels and is significantly correlated with clinical variables, including depressive symptom severity, episode recurrence, and suicide risk (25).

Evidence drawn from diverse regional contexts, including China, Brazil, Iran, Nigeria, and India, highlights a profound dual caregiving burden (26). Despite heterogeneous cultural backgrounds, these settings frequently share systemic constraints characterized by limited mental health resources and underdeveloped community care infrastructures, thereby necessitating a heavy reliance on family members as primary care providers (27). Furthermore, in many traditional and Asian contexts, strong cultural norms frame caregiving as a mandatory moral obligation, wherein patient recovery outcomes are closely tied to familial self-evaluation (28). It is hypothesized that such moral expectations might induce feelings of shame and guilt, which could exacerbate psychological distress among caregivers. Consequently, these individuals frequently experience a dual burden comprising insufficient objective healthcare support and significant subjective moral pressure. From a health economics perspective, the global economic value of informal caregiving exceeds the aggregate expenditures for formal institutional care services (29). Within a healthcare landscape marked by a shortage of psychiatric professionals and escalating medical costs, more than 60% of individuals with depression depend heavily on informal caregivers during both acute depressive episodes and long-term maintenance phases (30). Given the multifaceted characteristics of caregiving burden, a structured conceptual framework is required to systematically identify and synthesize its associated variables.

**Figure 1:**
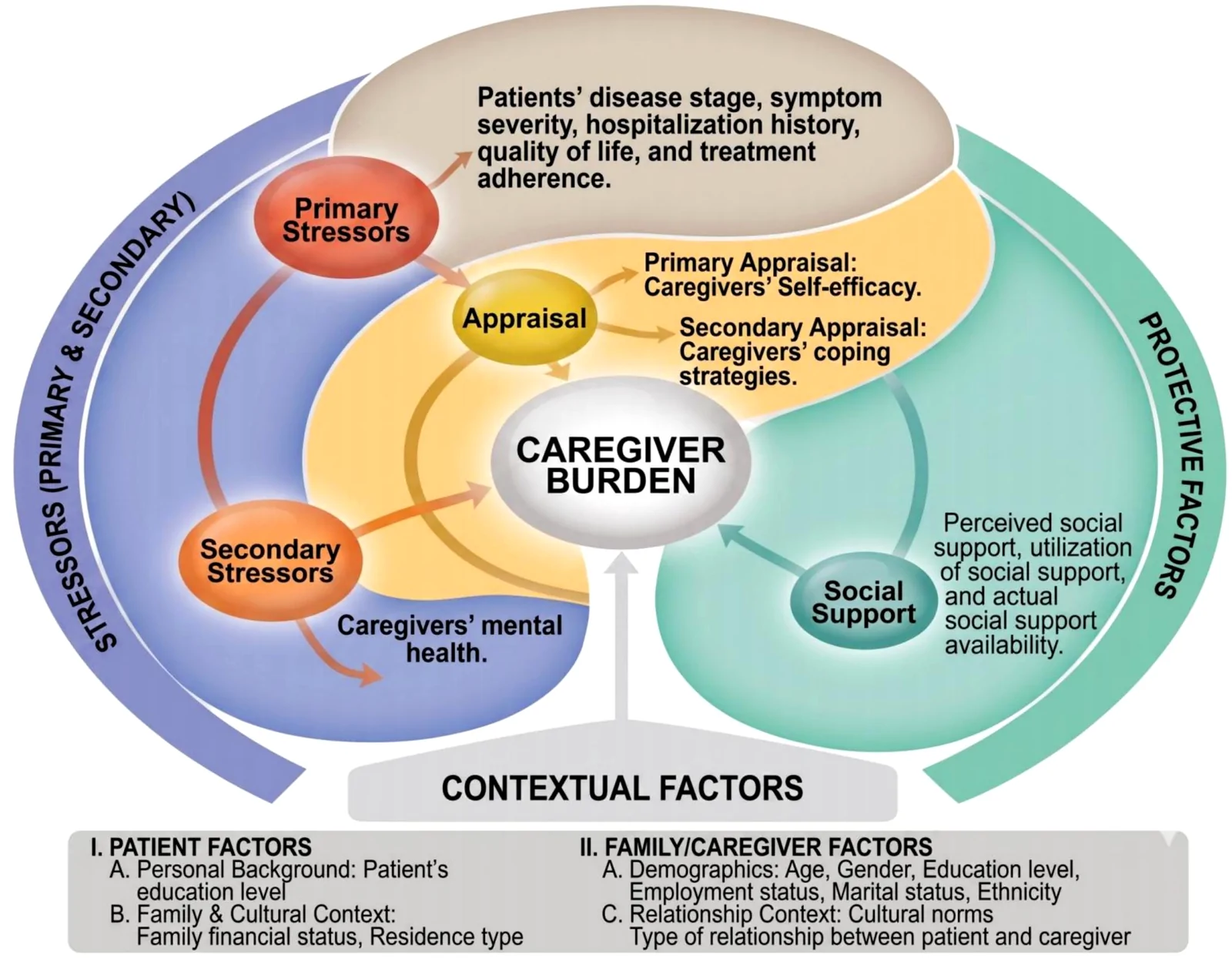
Stress-appraisal model based on Greenwell et al. (2015) (31).

The Stress-Appraisal Mode (**Figure 1**) provides a well-recognized theoretical foundation for investigating the caregiving experience (31). Rather than asserting direct causality, this framework clarifies how objective stressors, such as depressive symptom severity and caregiving hours, are dynamically associated with subjective cognitive appraisals, including self-efficacy and psychological resilience, as well as protective factors such as perceived social support (31–33). These appraisal and protective dimensions act as mediating variables that correlate with ultimate caregiver burden outcomes. The application of this theoretical model avoids the fragmentation of empirical research and provides a structured analytical perspective for interpreting dyadic interactions within the context of depression caregiving.

The current study was designed with two primary objectives. The first objective was to estimate the pooled mean score of caregiving burden among informal caregivers of patients with depression, utilizing standardized measurements derived from the 22-item Zarit Burden Interview (ZBI-22). The second objective was to systematically identify, categorize, and synthesize factors associated with this caregiving burden, guided by the Stress-Appraisal Model, thereby providing an evidence base for the development of targeted support strategies for caregivers of individuals with depression.

## Methods

### Protocol and registration

The protocol for this systematic review and meta-analysis is registered in the International Prospective Register of Systematic Reviews (PROSPERO) with the ID CRD420261373459. The current review used a Preferred Reporting Items for Systematic Reviews and Meta-Analyses (PRISMA) compliant methodology for searching the literature, selecting studies, extracting data, and reporting conclusions. To ensure full adherence and transparency, a completed PRISMA 2020 checklist is provided as **Supplementary Material**.

### Search strategy

The databases PubMed, Embase, MEDLINE, PsycINFO, Web of Science, CNKI, Wan fang, and VIP were used to search for research articles. Every database has a unique search method that mixes free-text terms with controlled vocabulary (Medical Subject Headings) related to depression, informal caregiver burden, and informal caregivers, combined using Boolean operators. The search for these articles was conducted from the inception of each database to January 5, 2026. The detailed search strategy and specific strings for each database are documented in the **Supplementary Material**. Additionally, the included studies’ reference lists were checked and manually screened to identify any further relevant articles. For studies involving multiple types of mental illnesses, we exclusively extracted data specifically related to caregivers of patients with depression (e.g., major depressive disorder).

### Eligibility criteria

#### Inclusion criteria

Articles that satisfy the following eligibility criteria are included in this review (1): all study participants are informal caregivers of individuals diagnosed with depression, (regardless of the disease stage and the caregivers’ duration of caregiving experience; (2) the study adopts an observational research design and investigates at least one potential correlate of caregiver burden among informal caregivers; (3) caregiver burden is quantified using the Zarit Burden Interview (ZBI-22) scale, and the study reports the mean value of ZBI-22 scores.

#### Exclusion criteria

Studies that did not specifically involve informal caregivers of patients with depression were disqualified. Additionally, studies whose complete outcome data regarding the burden on informal caregivers were not provided were not included.

### Data extraction

After the literature passed the inclusion screening and quality assessment, data extraction was performed via Microsoft Excel. Two authors, X.H and R.Z, independently extracted all required data from the included literature using a standardized extraction framework. The extracted fields covered: Authors, Publication Year, Country, Study Setting, Study Design, Study Population, Assessment Tool, Sample Size, Mean (SD), Burden Level, and Influencing Factors. Since none of the included studies were deficient in the required outcome measures, no request emails were sent to the original authors for supplementary data. Furthermore, this study pre-specified a contingency protocol for handling missing outcome data: in the event of missing key indicators during the data extraction phase, the corresponding authors would be contacted via email to request the original raw data; if supplementary data could not be obtained, effect sizes would be calculated using available statistical parameters; for datasets that are completely unsuitable for quantitative analysis, only qualitative description would be provided, and the robustness of the overall results would be verified via sensitivity analysis.

### Outcome measurements

This review aimed to ascertain the pooled mean score of caregiver burden among informal caregivers of patients with depression. STATA version 18.0 was utilized to calculate this overall pooled mean.

### Quality assessment

The Joanna Briggs Institute (JBI) critical appraisal checklists for analytical cross-sectional studies or cohort studies, as appropriate, were used to evaluate the methodological quality of the research papers included in this review (34). The potential risk of bias in study design, conduct, and analysis are the components that comprise this quality assessment tool. The original research quality was assessed by two reviewers independently using the JBI checklists. The instrument’s items are rated as “Yes,” “No,” “Unclear,” or “Not applicable,” accompanied by detailed guidance to assist reviewers in judging the presence of potential bias. If there were disagreements among the reviewers about the included studies’ quality evaluation, they were resolved through discussion. A summary of the quality assessment of the included studies is shown in the **Supplementary Material**. All cross-sectional studies used clear sampling strategies, provided detailed descriptions of the study participants and settings, and applied appropriate statistical analyses; however, two studies did not adopt adequate strategies to control for confounding factors (35, 36).

### Statistical procedure

Data extracted into Microsoft Excel were analyzed using STATA version 18.0. Characteristics of the primary studies were synthesized and presented through textual descriptions, tables, and forest plots. Ultimately, 11 studies were included in the analysis; all utilized the Zarit Burden Interview (ZBI) scale and reported quantitative data, which were employed to estimate caregiver burden levels (37). Pooled mean estimates were calculated and expressed as numerical values accompanied by 95% confidence intervals (CIs) and 95% prediction intervals.

We applied Cochran’s Q and *I*^2^ statistics to assess the original research for heterogeneity. The Der Simonian and Laird’s pooled effect of caregiver burden was estimated by using a continuous random-effects meta-analysis approach, which accounts for anticipated heterogeneity. To find out how a given group’s characteristics are associated with variations in the pooled burden, a subgroup analysis was carried out. To see how removing a single study result from the analysis affects the anticipated pooled mean score and its conclusion, we conducted a leave-one-out sensitivity analysis. If we found evidence of heterogeneity during analysis, we used the sensitivity analysis outcome to pinpoint its potential cause.

### Comprehensive framework of influencing factors based on the stress appraisal model

To systematically synthesize the complex interrelated factors identified in cross-sectional studies, a theoretical descriptive comprehensive analysis was conducted based on the stress assessment model. This analysis focused on five specific dimensions of the model: situational factors, stressors, appraisal factors, protective factors, and caregiver burden. Two researchers independently mapped the reported influencing factors from each study to these dimensions; discrepancies or ambiguities in classification were resolved by a third senior author.

## Result

### Search results

**Figure 2:**
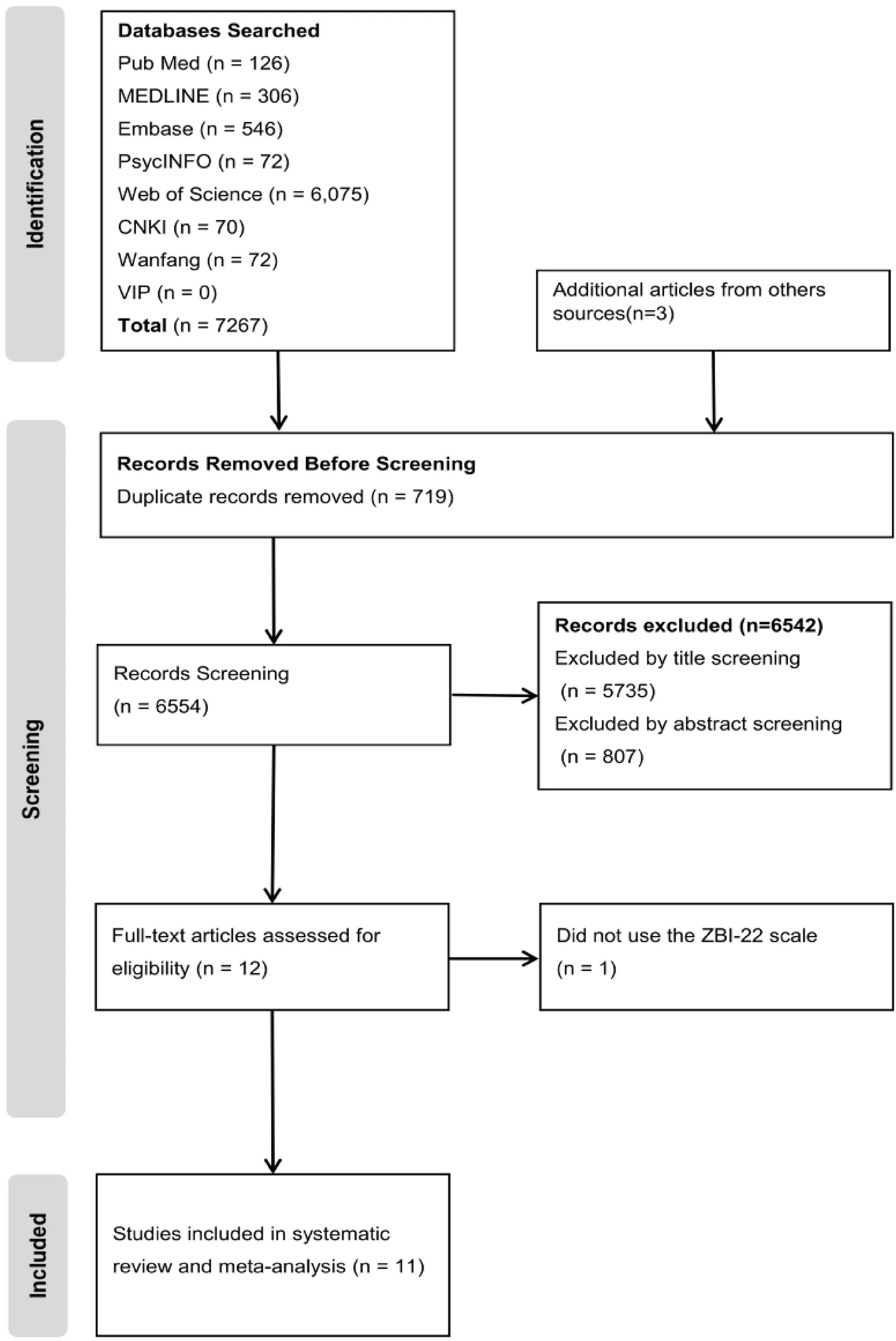
Flow diagram of the study selection process.

A total of 7,267 relevant articles were identified through comprehensive electronic database searches. Of these, 719 records were excluded due to duplication, and an additional 6,542 were discarded following title and abstract screening because full-text access was unavailable, study designs were incompatible, or the content was irrelevant to the review objectives. Full-text eligibility assessments were subsequently conducted for the remaining 12 articles. One study was excluded for not employing the ZBI-22 scale. Consequently, 11 studies meeting the inclusion criteria were included in this systematic review and meta-analysis (**Figure 2****).**

### Characteristics of included studies

This systematic review and meta-analysis incorporated 11 primary studies examining the burden levels and associated factors among informal caregivers of patients with depression. In terms of publication chronology, four studies were conducted in 2002, 2014, 2016, and 2018, respectively, whereas the remaining seven were published between 2021 and 2025. Geographically, the research spanned five countries, with six studies conducted in China (38–43), two in Nigeria (35, 44), and one each in Brazil (45), Iran (36), and India (46). The total sample size across all included studies comprised 1,366 respondents. The 22-item Zarit Burden Interview (ZBI-22) was consistently employed as the measurement instrument for assessing caregiver burden. All original studies included in this review utilized a cross-sectional design. Detailed characteristics of the included studies are summarized in **Table 1**.

**Table 1:** Characteristics of studies included in this systematic review and meta-analysis on caregiver burden among informal caregivers of patients with depression.

Authors	Publication year	Country	Study setting	Study design	Study population	Assessment Tool*	Sample size (n)	Mean (SD)	Burden level	Influencing factors
Shahbazi M	2025	Iran	Hospital-based	Cross sectional	Adults	(ZBI-22)	80	40.48 (16.19)	Moderate burden	Primary Stressors: Patients’ hospitalization history, Patients’ quality of life, Patients’ treatment adherence; Contextual Factors: Caregivers’ employment status, Family financial status, Patients’ education level
Wu Y	2025	China	Hospital-based	Cross sectional	Adolescents	(ZBI-22)	256	24.44 (12.07)	Mild burden	Secondary Stressors: Caregivers’ psychological health; Protective Factors: Caregivers’ resilience; Contextual Factors: Caregivers’ age, Caregivers’ gender, Type of relationship between patient and caregiver
Xin F	2025	China	Hospital-based	Cross sectional	Adults	(ZBI-22)	112	41.39 (9.93)	Moderate burden	Primary Stressors: Patients’ disease stage, Patients’ symptom severity; Protective Factors: Social support (perceived and received); Contextual Factors: Caregivers’ age, Caregivers’ education level, Caregivers’ gender, Family financial status; Social support significantly mediates the association between stressor exposure and informal caregiving burden
Shengxin D	2025	China	Hospital-based	Cross sectional	Adolescents	(ZBI-22)	333	53.17 (11.60)	Moderate burden	Secondary Stressors: Caregivers’ psychological health; Primary Appraisal: Caregiving self-efficacy; Contextual Factors: Caregivers’ education level, Caregivers’ gender, Family financial status
Shuyi L	2022	China	Hospital-based	Cross sectional	Adults	(ZBI-22)	180	33.53 (22.75)	Mild burden	Primary Appraisal: Caregiver involvement; Protective Factors: Social support (perceived and received); Contextual Factors: Caregivers’ education level, Caregivers’ employment status, Caregivers’ ethnicity, Caregivers’ gender, Caregivers’ marital status, Caregivers’ medical history, Cultural norms, Family financial status, Family residence type, Type of relationship between patient and caregiver
Xiangming L	2022	China	Hospital-based	Cross sectional	Adolescents	(ZBI-22)	76	42.17 (12.23)	Moderate burden	Primary Stressors: Patients’ disease stage; Contextual Factors: Caregivers’ education level, Caregivers’ employment status, Caregivers’ marital status, Type of relationship between patient and caregiver
Udoh EE	2021	Nigeria	Hospital-based	Cross sectional	Adults	(ZBI-22)	104	35.03 (14.67)	Mild burden	Secondary Stressors: Caregivers’ psychological health; Contextual Factors: Caregivers’ gender, Caregivers’ marital status, Patients’ medical history
Xin G	2018	China	Hospital-based	Cross sectional	Adolescents	(ZBI-22)	18	32.00 (15.00)	Mild burden	Primary Stressors: Patients’ disease stage, Patients’ quality of life, Patients’ symptom severity; Secondary Stressors: Caregivers’ psychological health; Contextual Factors: Caregivers’ age, Caregivers’ education level, Caregivers’ employment status, Caregivers’ gender, Family financial status
Kazhungil F	2016	India	Hospital-based	Cross sectional	Adults	(ZBI-22)	25	44.72 (15.07)	Moderate burden	Contextual Factors: Caregivers’ employment status, Family financial status, Patients’ education level, Type of relationship between patient and caregiver
Olawale KO	2014	Nigeria	Hospital-based	Cross sectional	Adults	(ZBI-22)	100	41.32 (9.82)	Moderate burden	Primary Stressors: Patients’ disease stage, Patients’ hospitalization history, Patients’ symptom severity; Secondary Stressors: Caregivers’ psychological health; Contextual Factors: Family financial status, Type of relationship between patient and caregiver
Scazufca M	2002	Brazil	Outpatient clinics	Cross sectional	Adults	(ZBI-22)	82	31.10 (15.70)	Mild burden	Primary Stressors: Patients’ disease stage, Patients’ symptom severity; Contextual Factors: Caregivers’ age, Caregivers’ gender, Type of relationship between patient and caregiver

NB: *: -Zarit Burden Interview (ZBI-22): Comprising 22 items with a total score ranging from 0 to 88, where higher scores indicate greater caregiver burden; typically, scores of 0–20 represent no burden, 21–40 indicate mild burden, 41–60 indicate moderate burden, and>60 indicate severe burden.

### Pooled mean of caregiver burden

**Figure 3:**
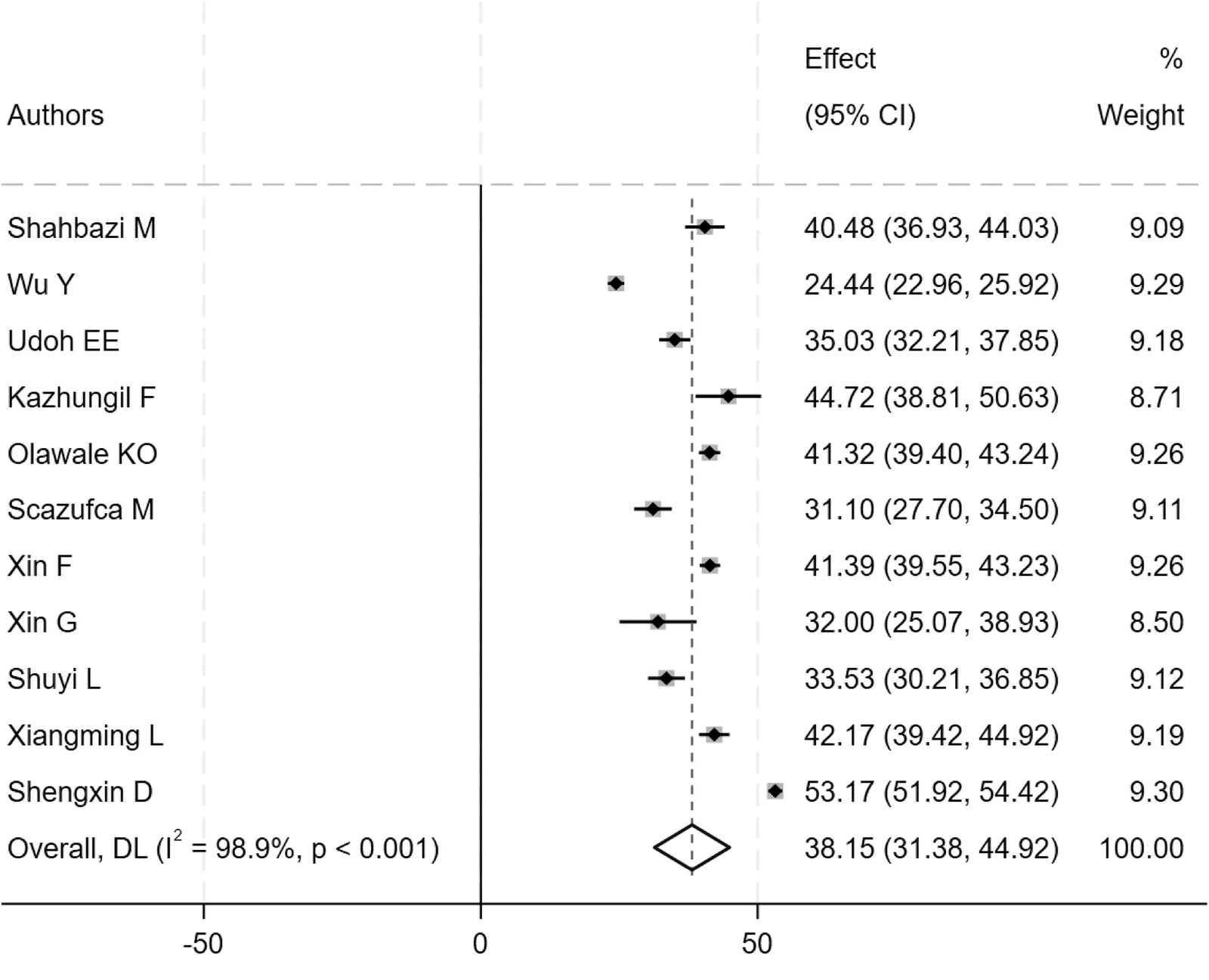
Forest plot showing the pooled mean of caregiver burden among informal caregivers of patients with depression. Weights are from random-effects model.

Eleven eligible studies were incorporated into this systematic review and meta-analysis to ascertain the pooled mean score of caregiver burden among informal caregivers of patients with depression. The overall pooled mean score was determined to be 38.15 (95% CI: 31.38, 44.92; *I*^²^ = 98.9%, *p* < 0.001), with a 95% prediction interval ranging from 19.67 to 56.67 (**Figure 3**).

### Heterogeneity and publication bias

**Figure 4:**
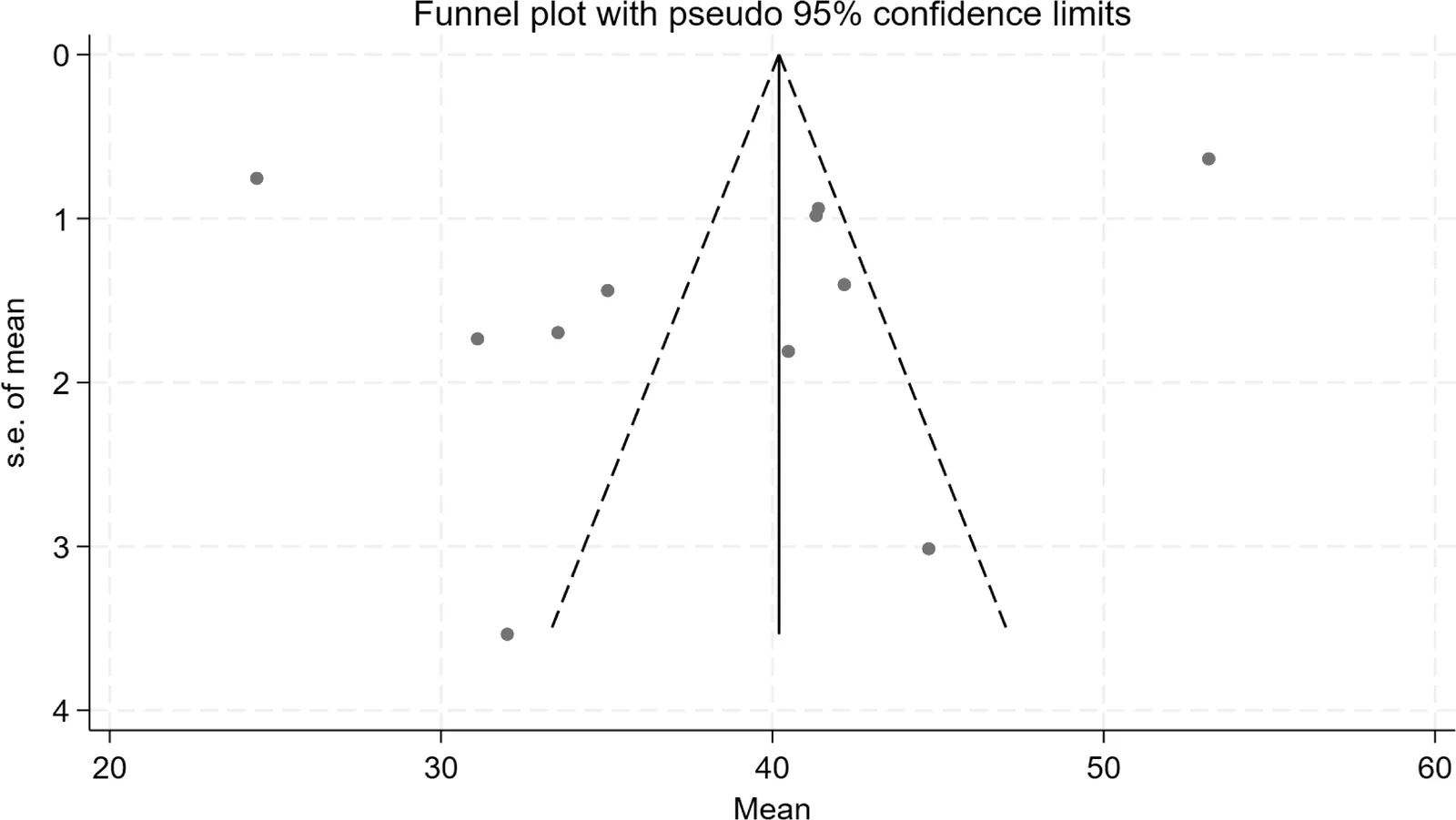
A funnel plot test of pooled mean of caregiver burden among informal caregivers of patients with depression.

The included papers in the current systematic review and meta-analysis had heterogeneity, as shown by the test statistics (*I*^2^; *I*^2^ = 98.9%, *P*-value < 0.001). To evaluate potential publication bias, two complementary methods were utilized. Visual inspection of the funnel plot revealed a symmetrical distribution of the included studies, indicating no evidence of publication bias (**Figure 4**). Furthermore, Egger’s test yielded a p-value of 0.498 (**Table 2**), thereby statistically confirming the absence of significant publication bias. No evidence of publication bias was detected; however, the small number of studies limits the power of formal tests.

**Table 2:** Egger’s test of caregiver burden among informal caregivers of patients with depression.

Std Eff	Coefficient	Std. err.	*t*	*P* > *t*	[95% conf. interval]
Slope	45.05523	7.661753	5.88	0.000	27.72314, 62.38732
Bias	-4.77046	6.763482	-0.71	0.498	-20.07052, 10.5296

### Subgroup analysis

A subgroup analysis was performed to explore potential sources of heterogeneity and to assess the variation in caregiver burden across different clinical and demographic contexts. The detailed results of this subgroup analysis are summarized in **Table 3**.

**Table 3:** Subgroup analysis of caregiver burden among informal caregivers of patients with depression.

Variables	Subgroup	Number of studies	Mean(95% CI)	*I* ^2%^	*p*-value
Country	China	6	37.85(26.72, 48.97)	99.4	<0.001
	Nigeria	2	38.26(32.10, 44.42)	92.3	<0.001
	Others*	3	38.52(30.67, 46.37)	90.8	<0.001
Patient population	adult	7	38.08(34.82, 41.34)	89.1	<0.001
	adolescent	4	38.00(20.96, 55.04)	99.6	<0.001
Severity of depression	moderate	2	35.51(22.74, 48.28)	98.7	<0.001
	severe	2	42.40(29.85, 54.94)	98.1	<0.001
Patient’s disease course	<1	2	34.42(27.84, 40.99)	94.3	<0.001
	>1	2	39.26(33.34, 45.18)	88.8	0.003

NB: *: -Brazil, Iran, India.

Across geographical regions, the pooled mean scores for caregiver burden demonstrated relative consistency. Investigations performed in China reported a mean score of 37.85 (95% CI: 26.72, 48.97), which was comparable to the estimates from Nigeria of 38.26 (95% CI: 32.10, 44.42) and other countries, including Brazil, Iran, and India of 38.52 (95% CI: 30.67, 46.37). Similarly, no substantial difference was observed regarding the patient population; the mean burden score for caregivers of adult patients of 38.08 (95% CI: 34.82, 41.34) was nearly identical to that of caregivers of adolescent patients of 38.00 (95% CI: 20.96, 55.04).

However, noticeable clinical variations emerged when stratifying by disease characteristics. The subgroup analysis revealed a higher pooled mean burden score among caregivers of patients with severe depression of 42.40 (95% CI: 29.85, 54.94) compared to those caring for patients with moderate depression of 35.51 (95% CI: 22.74, 48.28). Furthermore, caregivers of patients with a disease course exceeding one year experienced a higher mean burden of 39.26 (95% CI: 33.34, 45.18) than those caring for patients with a disease course of less than one year of 34.42 (95% CI: 27.84, 40.99).

It is important to note that the subgroup findings regarding Nigeria, severity of depression, and patient’s disease course should be interpreted with caution due to the limited number of included studies (n = 2) in these respective strata.

### Sensitivity analysis

A leave-one-out sensitivity analysis was conducted to explore potential sources of heterogeneity and assess the robustness of the findings. By systematically omitting one study at a time, we evaluated the influence of each individual dataset on the overall pooled mean score of caregiver burden. Because all recalculated point estimates fell within the 95% confidence intervals of the primary analysis, the data demonstrate that no single study significantly altered the overall burden level, confirming the stability of our synthesized results (**Table 4****).**

**Table 4:** Leave-one-out sensitivity analysis for caregiver burden among informal caregivers of patients with depression.

Study omitted	Estimate 95% CI	Heterogeneity	
		*I* ^2^	*P*-value
Shahbazi M	37.91 (30.63, 45.19)	99.0	<0.001
Wu Y	39.60 (34.41, 44.79)	97.6	<0.001
Udoh EE	38.46 (31.13, 45.79)	99.0	<0.001
Kazhungil F	37.52 (36.38, 40.66)	99.0	<0.001
Olawale KO	37.82 (30.11, 45.53)	99.0	<0.001
Scazufca M	38.85 (31.67, 46.64)	99.0	<0.001
Xin F	37.81 (30.05, 45.58)	99.0	<0.001
Xin G	38.72 (31.61, 45.82)	99.0	<0.001
Shuyi L	38.61 (31.37, 45.85)	99.0	<0.001
Xiangming L	37.74 (30.35, 45.13)	99.0	<0.001
Shengxin D	36.60 (31.48, 41.71)	97.3	<0.001

### Descriptive integration of the influencing factors based on the pressure assessment model

The analysis was conducted sequentially across model dimensions. Among the 11 included studies, nine addressed primary stressors: five examined the association between depression severity and caregiving burden (35, 39, 41, 43, 45), three investigated the relationship between disease duration and caregiving burden (39, 41, 43), and individual studies additionally assessed treatment non-adherence, age of onset of depression, and number of hospitalizations (35). All identified factors demonstrated positive correlations with caregiving burden.

Regarding secondary stressors, five studies investigated the relationship between caregivers’ psychological issues and caregiving burden (35, 40, 44, 45, 47). The assessed factors, including anxiety, associated stigma, social avoidance, and psychological distress, were all found to exhibit positive correlations with caregiving burden.

In the domain of primary appraisal, two studies examined the associations of self-efficacy and psychological resilience with caregiving burden (39, 47), both reporting negative correlations. Furthermore, a study demonstrated that psychological resilience mediates the relationship between stressors and caregiving burden (47).

For secondary appraisal, a single study assessed the relationship between negative coping strategies and caregiving burden, with results indicating a positive correlation (40).

Concerning protective factors, two studies reported on the correlations between the number of collaborative caregivers, perceived social support, utilization of social support resources, and caregiving burden (39, 42). All results demonstrated negative correlations. Specifically, a study reported that social support mediates the relationship between stressors and caregiving burden (39).

Contextual factors were reported in all 11 included studies. Eight studies investigated the correlation between gender and burden; four reported a positive correlation for female caregivers, one reported a negative correlation for male caregivers, and three showed no significant correlation. Four studies examined the relationship between caregiver age and burden, all reporting a positive correlation. Five studies investigated the correlation between household income and financial burdens, all demonstrating a negative relationship.

The reviewed corpus comprised 11 publications. Primary stressors were predominantly associated with patient depression severity (n=9) and disease duration (n=3), whereas secondary stressors most frequently pertained to caregiver mental health status (n=5). Although assessments of protective factors were less frequently reported, mediators linking stressors to caregiving burden were identified. Contextual factors were documented in all 11 included studies, with family economic status, caregiver gender, and age constituting the most prevalent dimensions. Detailed data are presented in **Figure 1**; **Table 1**.

## Discussion

The present systematic review and meta-analysis provide a comprehensive quantitative synthesis of caregiver burden among informal caregivers of patients with depression. Through the statistical aggregation of 11 observational studies spanning five countries and encompassing a total cohort of 1,366 caregivers, an overall pooled mean Zarit Burden Interview (ZBI-22) score of 38.15 (95% CI: 31.38, 44.92) was derived, accompanied by a 95% prediction interval ranging from 19.67 to 56.67. According to established interpretive criteria for the ZBI-22 instrument (48), this aggregated score approximates the clinical threshold separating mild-to-moderate burden from moderate-to-severe burden. However, given the limited number of eligible studies and the presence of substantial statistical heterogeneity (*I*^2^ > 90%), this pooled estimate should be regarded primarily as a baseline reference value rather than an absolute diagnostic consensus.

To contextualize the magnitude of this burden, an exploratory, indirect comparison was conducted against reported ZBI-22 baseline scores across other chronic and acute clinical cohorts. It should be emphasized that such cross-disease comparisons remain inherently indirect and must be interpreted with considerable caution, as the underlying comparative data originate from independent investigations characterized by inevitable variations in study design, sociodemographic profiles, cultural norms, and healthcare infrastructure.

Despite these methodological constraints, several clinical perspectives can be inferred from an indirect comparative analysis. Evidence derived from psychiatric outpatient settings indicates that the mean ZBI score among caregivers of individuals diagnosed with severe mental illnesses, predominantly schizophrenia, reaches 47.97 points (4), which noticeably exceeds the 38.15-point baseline identified in the current investigation. This variation might be attributed to the distinct clinical manifestations of schizophrenia, which frequently involve positive symptoms, such as hallucinations and delusions, as well as severe social functional regression. Consequently, caregivers of patients with schizophrenia are frequently required to manage unpredictable behavioral agitation while navigating profound social stigma. In contrast, individuals suffering from depression generally preserve their capacity for reality testing and exhibit fewer outwardly disruptive behaviors. Therefore, a probable explanation for the lower burden score observed in depression caregiving is that the daily care demands do not reach the behavioral extremes typically associated with psychotic disorders. These literature-based observations suggest that variations in caregiver burden scores are likely correlated with the distinct clinical trajectories and symptomatology of specific psychiatric conditions.

When evaluating physical disabilities, a recent investigation conducted in China reported a mean ZBI score of 37.17 points among caregivers of young and middle-aged stroke survivors presenting with moderate-to-severe functional impairments (49). This figure is comparable to the 38.15-point mean observed in the present review. Although direct clinical equivalence cannot be established without controlled head-to-head investigations, this parallel suggests that caring for an individual with depression, who generally retains independent activities of daily living yet experiences profound affective distress and potential suicide risk (50), is associated with a level of subjective psychological distress comparable to that experienced by caregivers of hemiplegic stroke survivors requiring continuous physical assistance. A possible justification for this similarity is that the psychological strain of managing affective withdrawal and safety concerns in depression rivals the physical exhaustion associated with functional caregiving, thereby highlighting the clinical significance of the invisible burden inherent in psychiatric conditions (36).

Furthermore, a divergence in caregiving burden is observed when comparing the present findings with literature focusing on family caregivers of patients with terminal cancer receiving palliative care. Research from the Eastern Mediterranean region reported a mean ZBI score of 23.4 points in end-stage oncology settings (51), a value substantially lower than the pooled estimate calculated in this study. This numerical discrepancy must be interpreted prudently, as major sociocultural, religious, and healthcare support differences act as confounding variables. From a clinical perspective, a possible justification for this difference is that caregiving objectives during palliative oncology are often highly defined, such as somatic pain alleviation, and caregivers frequently receive structured support from multidisciplinary palliative teams alongside overt emotional gratitude from patients. Conversely, the core symptom constellation of depression involves social withdrawal, affective flattening, and anhedonia. It is plausible that informal caregivers of individuals with depression experience a lack of positive emotional reciprocity, which is frequently linked to feelings of relational isolation. This potential depletion of interpersonal coping resources, potentially co-occurring with maladaptive cognitive appraisals, might theoretically account for the elevated burden scores observed among depression caregivers compared to certain oncological cohorts (50).

When these exploratory observations are synthesized alongside broader psychiatric literature, it is suggested that caregivers’ ZBI scores are positively correlated with their own depressive symptomatology (35). When caregiving burden approaches the pooled estimate of 38.15 points identified in this review, the psychological resilience of informal caregivers faces substantial strain. This association points to a heightened vulnerability to a theoretical adverse cycle involving patient depression, caregiver exhaustion, and subsequent patient relapse. Consequently, it is recommended that clinical management strategies transition from an exclusively patient-centered framework toward an integrated, dyadic patient-family intervention model (38).

Although substantial statistical heterogeneity was detected among the primary studies (*I*^2^ > 90%), quantitative aggregation was deemed appropriate to identify overarching analytical trends, supported by a structured narrative synthesis based on the standardized application of the ZBI scale across all included literature. Subgroup analyses revealed that heterogeneity remained elevated across most strata, which might be explained by the presence of unmeasured clinical and contextual confounding factors that could not be adequately controlled through statistical stratification. These uncontrolled variables likely include variations in familial roles and caregiving structures, as primary studies frequently combined parents, spouses, and adult children into a single sample despite distinct emotional and financial dynamics. This methodological limitation likely contributed to the wider confidence intervals observed in the adolescent subgroup. Furthermore, substantial disparities in caregiving contexts, such as differences in clinical treatment phases, daily caregiving hours, and access to formal social support, might also account for the residual heterogeneity. These findings further confirm that reported informal caregiving burden levels are sensitive to, and closely associated with, diverse clinical and socio-environmental contexts. Given the limited number of studies available within individual subgroups, these stratified trends should be interpreted with appropriate scientific caution.

Finally, the incorporation of the Stress-Appraisal Model (52) provided a structured theoretical framework to systematically categorize the multidimensional correlates of caregiver burden among individuals managing depression. The comprehensive descriptive synthesis indicated that existing empirical literature has predominantly concentrated on primary stressors and secondary caregiver psychological distress (52). Conversely, the potential buffering effects of coping strategies and formal social support mechanisms remain substantially under investigated.

Because quantitative validation of direct causal pathways was precluded by the cross-sectional design of the primary studies, this descriptive synthesis establishes a preliminary, evidence-informed framework for applying stress-appraisal concepts within depression caregiving research. Future investigations should progress beyond cross-sectional correlation identification by employing longitudinal, prospective cohort, or time-series designs. Such methodologies are required to examine the dynamic, temporal relationships between objective stressors, mediating appraisal mechanisms, protective resources, and subsequent caregiving burden outcomes in clinical settings.

### Limitations

Several methodological limitations should be acknowledged when interpreting the findings of this systematic review and meta-analysis. First, the relatively limited number of eligible primary studies (n=11) and the substantial statistical heterogeneity observed across the literature necessitate that the overall pooled mean score be treated primarily as a reference estimate, with actual caregiver burden levels requiring individualized clinical assessment. Second, the underlying evidence base was derived exclusively from cross-sectional study designs. Although significant statistical associations between various theoretical model dimensions and caregiver burden were successfully synthesized, the absence of longitudinal or cohort investigations precludes any definitive assertions regarding causal inference or temporal directionality. Finally, to maintain measurement consistency, quantitative inclusion criteria were restricted to studies utilizing the ZBI-22 instrument. While the ZBI-22 represents a widely validated clinical tool for assessing caregiver burden, this restriction inevitably excluded relevant studies utilizing alternative assessment instruments, thereby narrowing the psychometric scope of the synthesis.

## Conclusion

The current systematic review and meta-analysis showed that caregivers of patients with depression generally experience substantial caregiving burden, with pooled effect estimates indicating that the overall severity of this burden approaches the moderate level. Nevertheless, due to the limited quantity of caregiving burden is affected by multiple clinical variables. Accordingly, the findings of current systematic reviews should be interpreted with caution. Furthermore, this study proposes that public health interventions targeting mental health indicators and social support systems should be implemented for informal caregivers of individuals with depression, with the objectives of reducing the caregiving burden of this population and potentially improving the quality of care accessed by patients with depression.

## Data Availability

The original contributions presented in the study are included in the article/**Supplementary Material**. Further inquiries can be directed to the corresponding authors.
